# Concurrent Renorrhaphy During Renal Mass Excision in Laparoscopic Nephron-Sparing Surgery: A Novel Surgical Technique

**DOI:** 10.5152/tud.2025.24062

**Published:** 2025-01-03

**Authors:** Nurullah Hamidi, Tuncel Uzel

**Affiliations:** Department of Urology, University of Health Sciences, Dr. Abdurrahman Yurtaslan Ankara Oncology Training and Research Hospital, Ankara, Türkiye

## Objective

Laparoscopic nephron sparing surgery (NSS) can be performed by mainly 2 methods, off-clamp or on-clamp. Continuous bleeding during the off-clamp method may impair the clear visualization of the border between the tumor and parenchyma, even though it is done safely in experienced hands. Therefore, some surgical modifications may be needed during mass excision and renorraphy.

In this video brief, we aimed to present a new off-clamp NSS technique and our case series. The difference of this technique from others is that renorrhaphy was performed during mass excision concurrently to reduce the amount of bleeding.

## Materials and Methods

Laparoscopic transperitoneal NSS was performed on a 40-year-old male patient with a low-complexity lower pole mass (2.5 × 2 cm) in the right kidney, characterized by a RENAL nephrometry score of 4p.

On left lateral decubitus position, after port placement, the ascending colon was medialized and the right ueter was found. The renal pedicle was dissected, and the main renal artery was secured with the vessel loop. After opening Gerota’s fascia, the mass was found on the lateral side of lower pole. The excision margin was determined by cautery. A 3-0 V-Loc suture was fixed to the anterior abdominal wall just before mass excision. Along the margin, the renal parenchyma was incised by scissors at a depth of 3-4 mm. Excision continued until there was enough place to pass the suture. Then renorrhaphy was started with the V-Loc suture to reduce bleeding. Suturing continued until reaching to excision limit. Excision continued until serious bleeding occurred. If serious bleeding occurred, suturing was performed again. If there was no bleeding, excision was completed after controlling the tumor base with suturing. Intraperitoneal air pressure was reduced to detect hidden bleeding. Anti-bleeding powder was applied to the excision area. Written informed consent was obtained from the patients who agreed to take part in the study.

## Results

The total operative and renorraphy time was 85 and 10 minutes, respectively. The bleeding amount was 150 mL. The pathologic report confirmed a 2.5 × 2 × 2 cm clear cell renal cell carcinoma. No major complications were observed during surgery. After 46 months, there is no local recurrence or metastasis.

### Results of Case Series

We performed 10 NSS surgeries using the technique we described. All the surgeries were performed by a single surgeon (N.H.). The mean age, largest tumor diameter, and RENAL nephrometry score of our patients were 56.5 ± 1.87 years,22.8 ± 4.3 mm, and 4.7 ± 1, respectively. Tumor localizations were in the lower and middle pole in 8 and 2 patients, respectively. All the tumors were localized on the anterior and/or lateral side of the kidneys. The mean total operation time and mean excision-renorrhaphy time were 100 ± 15.8 and 16 ± 5.3 minutes, respectively. The mean estimated blood loss and hemoglobin drop during surgery were 233 ± 57 mL and 1.1 ± 0.3 g/dL, respectively. None of the patients required erythrocyte replacement, and no major complications were detected during the operation. The mean hospital stay and drain removal time were 2.5 ± 0.7 and 1.7 ± 0.6 days, respectively. Histopathological examination revealed clear cell and papillary type renal cell carcinoma in 7 and 3 patients, respectively. All patients had negative surgical margins. The mean follow-up was 29.7 ± 9.5 months. Metastasis or recurrence did not occur during the follow-up period in any of the patients. In examining the estimated glomerular filtration rate (eGFR) before surgery and at the time of the last visit, it was found that eGFR levels decreased by an average of 5.27 ± 2 mL/min/1.73 m^2^.

## Conclusion

In the novel technique we presented, as we performed tumor excision and renorrhaphy concurrently, we believe that this approach significantly reduced bleeding from the tumor bed during excision. In selected cases (tumors with small sizes, low complexity, and highly exophytic characteristics), we believe that laparoscopic nephron-sparing surgery can be performed without increasing blood loss, without clamping the renal vessels, and without the use of costly additional surgical instruments such as bulldog clamps, thanks to this technique.

However, we recommend caution when applying this technique to large, complex, or cystic tumors. Compared to on-clamp techniques, this approach may lead to bleeding during tumor excision, which can obscure the boundary between the tumor and normal renal parenchyma. The resulting loss of anatomical clarity increases the risk of rupture, particularly in cystic tumors. Ruptured cystic masses may pose a risk of local recurrence or peritoneal carcinomatosis during follow-up.

## Data Availability

The data that support the findings of this study are openly available at http://DOI:10.5152/tud.2025.24062.

